# Supplementary material for: The Effects of Catabolism Relationships of Leucine and Isoleucine with BAT2 Gene of Saccharomyces cerevisiae on High Alcohols and Esters
Source: Genes (Basel). 2022 Jun 30;13(7):1178. doi: 10.3390/genes13071178 (PMC9321263; doi:10.3390/genes13071178)
Supplement: Supplementary file 1 [file genes-13-01178-s001.zip › genes-1655968-supplementary.pdf]

Supplementary Materials

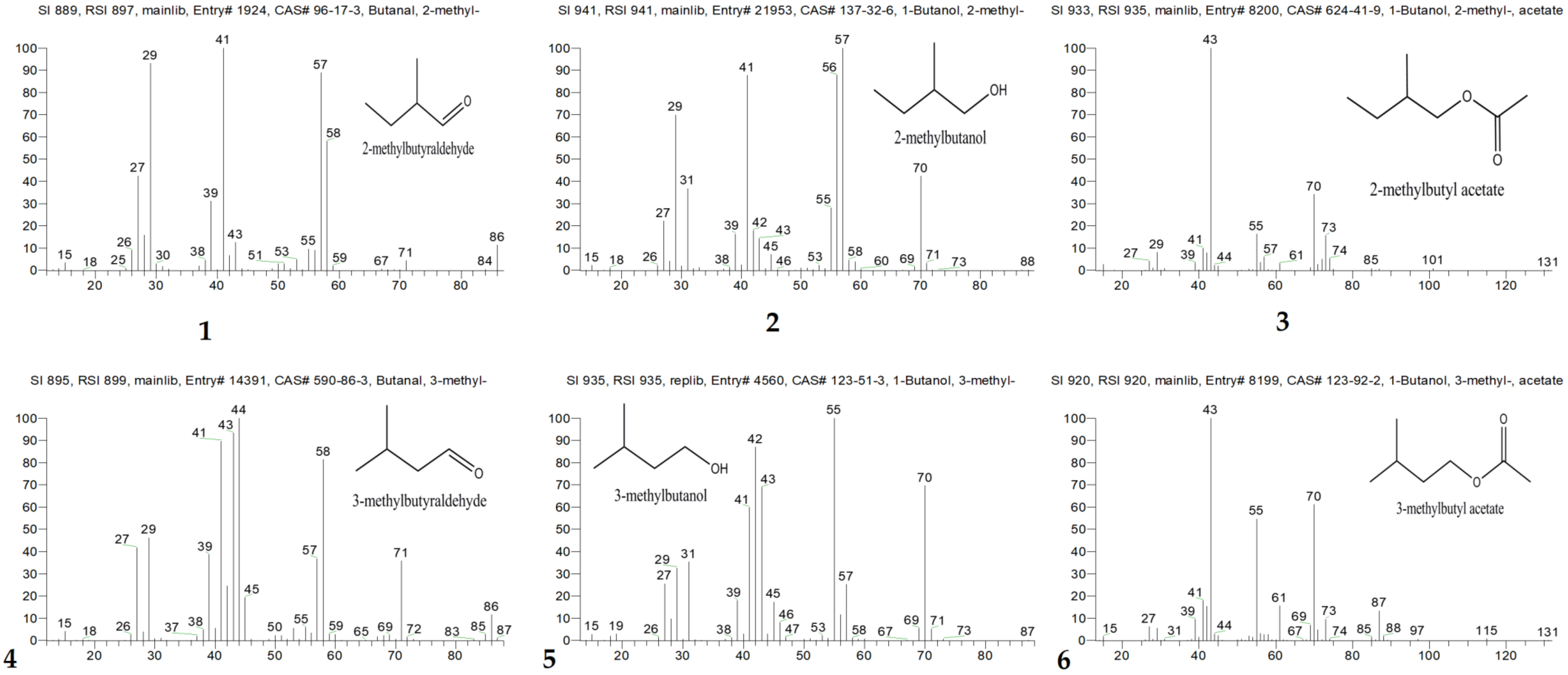

Figure S1. The mass spectrograms of the different branched-chain alcohols and easts products converted by Strain 38(*ΔBAT2*) and Parental strain 38 used Ile or Leu as sole nitrogen source. (1), (2), (3) represents the mass spectrogram of 2-methylbutyraldehyde, 2-methylbutanol, 2-methylbutyl acetate derived from the addition of Ile, respectively. (4), (5), (6) represents the mass spectrogram of 3-methylbutyraldehyde, 3-methylbutanol, 3-methylbutyl acetate derived from the addition of Leu, respectively.

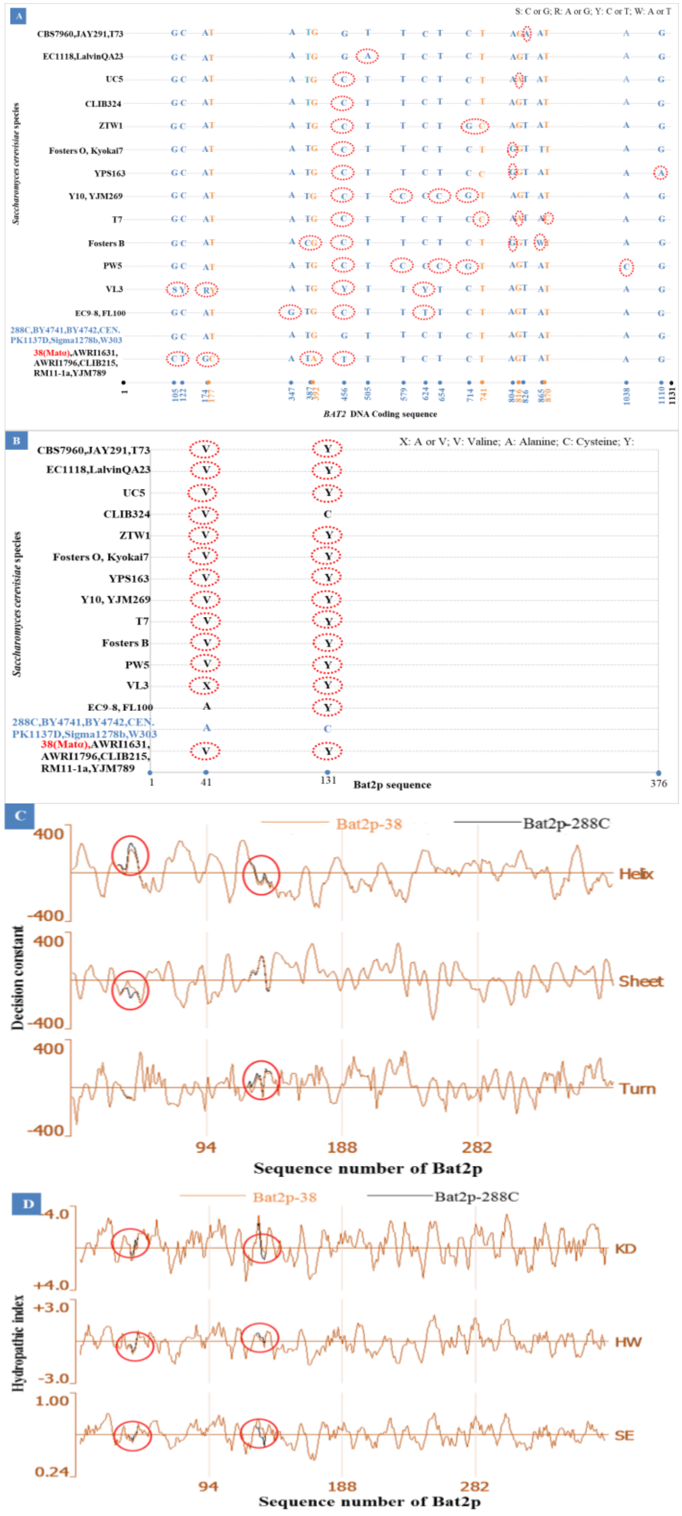

A
S: C or G; R: A or G; Y: C or T; W: A or T
CBS7960,JAY291,T73
EC1118,LalvinQA23
UC5
CLIB324
ZTW1
Fosters O, Kyokai7
YPS163
Y10, YJM269
T7
Fosters B
PW5
VL3
EC9-8, FL100
288C,BY4741,BY4742,CEN.PK1137D,Sigma1278b,W303
38(Mato),AWRI1631,AWRI1796,CLIB215,RM11-1a,YJM789
Saccharomyces cerevisiae species
BAT2 DNA Coding sequence
B
X: A or V; V: Valine; A: Alanine; C: Cysteine; Y:
Bat2p sequence
C
Bat2p-38
Bat2p-288C
Helix
Sheet
Turn
Decision constant
Sequence number of Bat2p
D
KD
HW
SE
Hydropathic index

Figure S2. The structural features of *BAT2* gene and BAT2p of *S. cerevisiae* strains**.** Bat2p-38 is the expression protein sequence of *BAT2* gene in parental *S. cerevisiae* 38. Bat2p-288c is the expression protein sequence of *BAT2* gene in wildtype S. cerevisiae 288C. KD means Kyte and Doolittle method. HW means Hopp and Woods method. SE means surface exposure. *BAT2* gene: 1131bp; Bat2p: 376 Amino acid residues.
